# Supplementary material for: Causal interplay between lactose intolerance and gut microbiota: a combined bidirectional Mendelian randomization and in vivo validation study
Source: Front Nutr. 2026 Jun 1;13:1803337. doi: 10.3389/fnut.2026.1803337 (PMC13265576; doi:10.3389/fnut.2026.1803337)
Supplement: Supplementary file 1 [file Data_Sheet_1.zip › supplementary materials/Forward/leave one out cross validation/ebi-a-GCST90027696.finngen_R12_E4_LACTONAS.pdf]

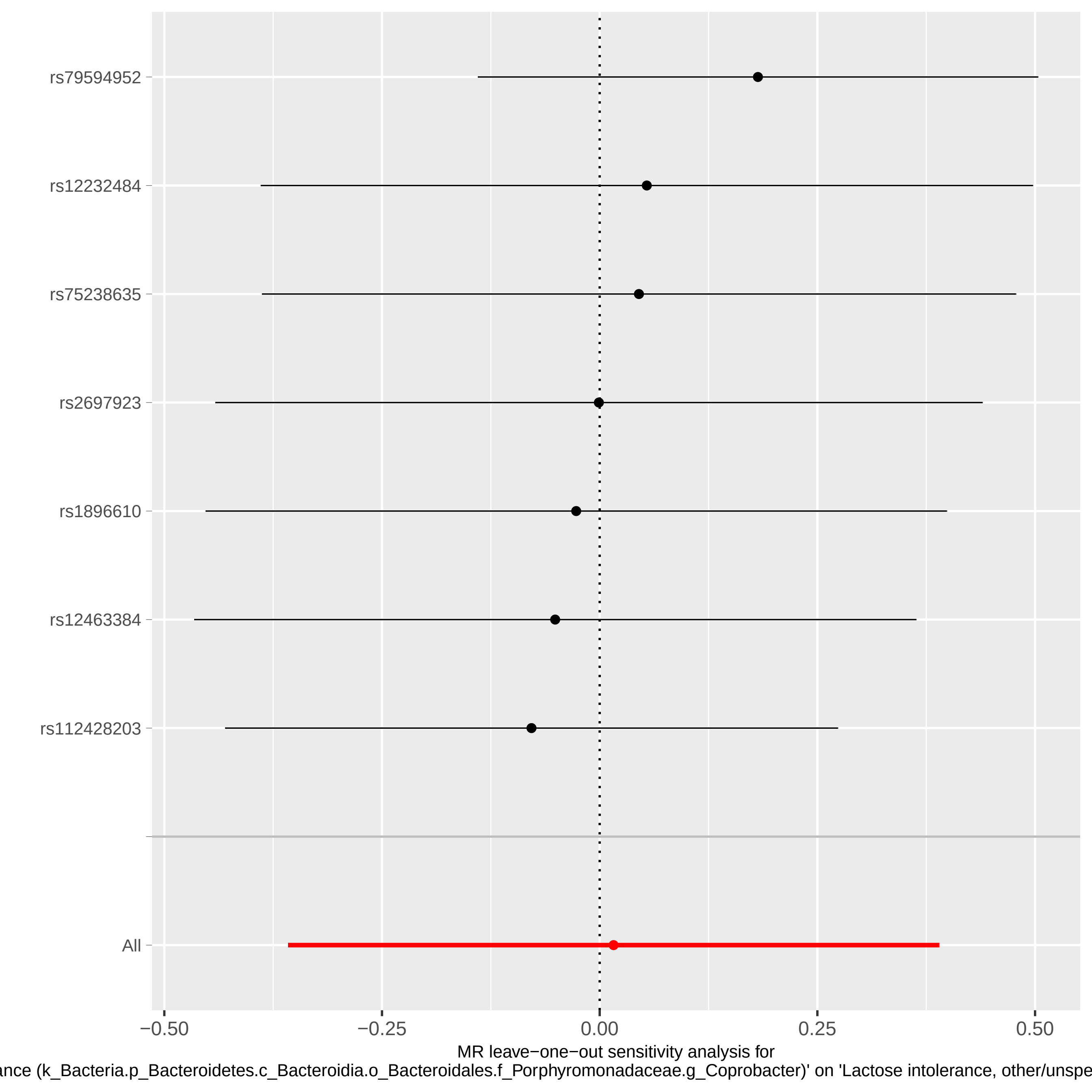

rs79594952
rs12232484
rs75238635
rs2697923
rs1896610
rs12463384
rs112428203
All
−0.50
−0.25
0.00
0.25
0.50
MR leave−one−out sensitivity analysis for
ance (k_Bacteria.p_Bacteroidetes.c_Bacteroidia.o_Bacteroidales.f_Porphyromonadaceae.g_Coprobacter)' on 'Lactose intolerance, other/unspe
